# Acoustic Impulsive Noise Based on Non-Gaussian Models: An Experimental Evaluation

**DOI:** 10.3390/s19122827

**Published:** 2019-06-25

**Authors:** Danilo Pena, Carlos Lima, Matheus Dória, Luan Pena, Allan Martins, Vicente Sousa

**Affiliations:** 1 Department of Electrical Engineering, Federal University of Rio Grande do Norte, 59078-970 Natal, Brazil; luan.gppcom@gmail.com (L.P.); allan@dca.ufrn.br (A.M.); 2 Department of Communications Engineering, Federal University of Rio Grande do Norte, 59078-970 Natal, Brazil; carloslima@ufrn.edu.br (C.L.); matheusf@ufrn.edu.br (M.D.); vicente.sousa@ufrn.edu.br (V.S.)

**Keywords:** noise estimation, non-Gaussian noise, maximum likelihood, acoustic channel

## Abstract

In general, acoustic channels are not Gaussian distributed neither are second-order stationary. Considering them for signal processing methods designed for Gaussian assumptions is inadequate, consequently yielding in poor performance of such methods. This paper presents an analysis for audio signal corrupted by impulsive noise using non-Gaussian models. Audio samples are compared to the Gaussian, α-stable and Gaussian mixture models, evaluating the fitting by graphical and numerical methods. We discuss fitting properties as the window length and the overlap, finally concluding that the α-stable model has the best fit for all tested scenarios.

## 1. Introduction

The acoustic channel has received much attention in recent years due to many practical applications and some emerging technologies, such as speech recognition, smart speaker, sound source tracking and assistive technologies. It is known that the audio signals reception is degraded by the presence of undesirable interference caused by noise from vehicles, crowds, wind, machines and human-made audio noise.

One way to describe the acoustic noise characteristics is based on the use of probability distributions such as the α-stable, Gaussian mixture, Bernoulli-Gaussian, Poisson–Gaussian, hidden Markov model, among others. In this paper, we use the α-stable model and Gaussian mixture distributions because they are the most frequently used distributions employed to model impulsive noise. Especially for signal processing methods that rely on second-order statistics [1], the Gaussian assumption of acoustic noise behavior derives meaningful degradation or does not work well. Thus, the adoption of suitable models must be considered to reach accuracy and acceptable performance for these solutions. Although there are some studies on more realistic noise model with non-Gaussian distributions [2], few signal processing solutions have been established compared to those with Gaussian assumption.

The analysis of impulsive noise characteristics can be found for acoustic and non-acoustic channels. Overview of wireless communication solutions considering impulsive noise are presented in [3,4], including specific protocols and transmission mediums such as power line communications [5], underwater environment [6], and satellite communications [7]. However, their noise data and analysis are not able to generalize the model and characteristics to an audible acoustic channel. Depending on the carrier frequency and the transmission medium, different interferences are presented, and different conclusions may be achieved. Therefore, complementary works towards the modeling and estimation of audible acoustic channels in the presence of impulsive noise by non-Gaussian models are essential to contribute to the near future solutions in acoustic.

There is a considerable number of estimators for the chosen models. For α-stable distribution, there is an usual method that uses a plot to observe the data on a logarithm scale [8]. This method is empirical; hence, it is not always reliable. Another conventional method, found in distinct areas of studies [9], is based on quantiles of the stable distribution [10]. For a particular case, when the distribution is symmetric, a method based on the fractional moments is an alternative [11]. Several works use the Maximum Likelihood Estimator (MLE) due to the feasibility of its estimation, even for large data sets [12,13]. Additionally, there is a fast algorithm to compute the MLE, and it achieves the Cramér-Rao bound [14]. For these reasons, the MLE is used as the estimator for studies herein presented.

Several research groups have been working with Gaussian Mixture Model (GMM) to model audio signals [15]. They claim that the source noise is not only originating from a unique source (i.e., electromagnetic interference). For instance, the audio message can be corrupted by vehicle noise or unknown speakers. Each undesirable source (noise source) is modeled by a single class noise or an individual Gaussian from the GMM. Thus, besides the α-stable, we also consider the Gaussian Mixture Model (GMM) as a candidate noise model.

The analysis and investigation of impulsive noise in acoustic channels are still challenging, allowing some rooms for research opportunities. First, several commercial products, using traditional signal processing solutions, are emerging and subject to severe acoustic scenarios such as smart speakers [16,17], robotics [18] and video conference systems [19]. Second, researches have shown methods for non-Gaussian noise without justifying the real occurrence of these cases. Moreover, few works have exposed a qualitative analysis among the non-Gaussian models that include experimental validation, and the identification of real-life scenarios for each model. Finally, little effort has been presented to assess different acoustic scenarios over the same unified quantitative analyses.

This paper offers an evaluation of some acoustic scenarios using an experimental setup. First, two indoor environments are examined with less impulsiveness. Then, one outdoor scenario with severe impulsiveness is analyzed. We propose to analyze the fitting for the Gaussian, GMM, and α-stable models for all scenarios. We present graphical and numerical evaluation, addressing their properties, disadvantages, and advantages.

This paper is organized as follows. Section 2 presents the specificities of parameter estimation for non-Gaussian models related to impulsive noise characterization. We then introduce our experimental setup on Section 3, where measurements situations are described. The evaluation scenarios are defined on Section 4, along with the presentation and evaluation of the results. Finally, conclusions are summarized on Section 5.

## 2. Parameters Estimation

We use the Maximum Likelihood Estimation to determine the parameters of the non-Gaussian models (GMM and α-stable) of the collected data. This Section presents the main distribution parameters and how we applied the MLE to its estimation.

### 2.1. GMM Parameters Estimation

The GMM is a linear combination of Gaussians functions where the sum of all weight coefficients is equal to one. Thus, a random variable *y* with GMM distribution is defined by

(1) $$p\left(y\right)=\sum_{i=1}^{M}c_{i}N\left(x_{i}|\mu_{i},\sigma_{i}\right),\;\mathrm{with}\;\sum_{i}^{M}c_{i}=1,$$

where $c_{i}$ is the weight of the i-th Gaussian distribution function, *M* represents the number of Gaussian distributions in the mixture, and $N(x_{i}|\mu_{i},\sigma_{i})$ is a Gaussian distribution function given by

(2) $$N\left(x_{i}|\mu_{i},\sigma_{i}\right)=\frac{1}{\sqrt[{}]{2\pi }\sigma_{i}}e^{-\frac{\left(x_{i}-\mu_{i}\right)}{2\sigma_{i}^{2}}},$$

where $x_{i}$ is the i-th Gaussian random variable with its expected value $\mu_{i}$ and standard deviation $\sigma_{i}$ [20].

The MLE method is a way to estimate the parameters θ which specify a probability function $f(x_{i}|\theta )$ of a random variable *X* [21]. The estimation is based on the independent and identically distributed (i.i.d.) samples $x_{i}$ (observations) from the distribution, and a log-likelihood function, which is given by

(3) $$\ell \left(\theta \right)=\sum_{i=1}^{N}\log f\left(x_{i}|\theta \right).$$

Thus, the MLE chooses the model parameters $\hat{\theta }$ that maximize the likelihood function, yielding the most likely parameters to generate the observed data [22].

The Expectation-Maximization (EM) is a method to determine the MLE of the parameters $\theta_{GMM}$ of a GMM [23]. Therefore, assuming that the observed data is generated by *M* Gaussians, the estimated parameters $\theta_{GMM}$ are $\left(\mu_{i},\sigma_{i}^{2},c_{i}\right)$ for the whole set of *M* Gaussians.

The EM algorithm employs an iterative procedure that is obtained by alternating the expectation step (E-Step) with the maximization step (M-Step). In the E-Step, we calculate the expected value of the log-likelihood concerning the current estimate of the distribution (estimation of $\mu_{i}$ and $\sigma_{i}^{2}$ parameters). In the M-Step, we maximized these expected parameters of the E-Step, also improving the estimation of $c_{i}$. These parameters are then used to determine the new parameters in the next E-Step until convergence is detected [24].

We selected the initial parameters using a heuristic to find centroid seeds based on k-means, and the algorithm iterates over the steps until convergence.

### 2.2. α-Stable Parameters Estimation

Theoretical reasons for statistical modeling using α-stable distributions are based on the Generalized Central Limit Theorem and the stability property [8]. The Generalized Central Limit Theorem states that if the sum of i.i.d. random variables with or without finite variance converge, the limit distribution must be α-stable. According to the stability property, α-stable distributions are closed under convolution, i.e., the sum of two independent random variables with the same characteristic exponent is also α-stable, keeping the same characteristic exponent [2]. The third reason for using this model is that the measured data exhibits heavy tails and skewness. This behavior may come from a combination of different random variables, which justify the usage of α-stable model by Generalized Central Limit Theorem.

There are different parametrizations of α-stable distribution for different specifications of the characteristic function. We assume the parameters $\theta_{\alpha }=\left(\alpha ,\beta ,\gamma ,\delta \right)$ and the following characteristic function [2]:

(4) $$\begin{array}{c} \varphi \left(\omega ;\theta_{\alpha }\right)=\exp(-\gamma^{\alpha }{\left|\omega \right|}^{\alpha }\left[1-j\Theta \left(\omega ;\alpha ,\beta \right)\right]+j\delta \omega ) \end{array},$$

with

(5) $$\Theta =\left\{\begin{array}{cc} \beta \left(\tan\frac{\pi \alpha }{2}\right)\left(\mathrm{sign}\;\omega \right), & \;\alpha \neq 1\\ -\beta \frac{2}{\pi }\left(\ln|\omega |\right), & \;\alpha =1, \end{array}\right.$$

where

α: is the *characteristic exponent* such that 0<α<2,

β is the symmetry parameter such that −1≤β≤1,

γ is the dispersion or scale parameter such that γ>0,

δ is the location parameter such that −∞<δ<∞.

Finally, we also assume a Symmetric α-Stable (SαS) class, because it has proved to be very useful in modeling impulsive noise [11]. For such distribution class, β = 0 and δ = 0 [8].

Considering MLE for α-stable distribution, the fundamental issue is that there is no known a general closed formula for the probability density. Only when α has a specific value, there are expressions for densities. This is a problem regarding the calculation of the log-likelihood function in Equation (3).

However, we apply the direct integration method, detailed in [25], to estimate α and γ parameters by MLE for the SαS model, with the characteristic function described as

(6) $$\begin{array}{c} \varphi \left(\omega ;\alpha ,\gamma \right)=\exp(-\gamma^{\alpha }|\omega {|}^{\alpha }). \end{array}$$

The primary parameter, α, describes the heaviness of the distribution tails. The smaller the α, the heavier are the tails; thus, more impulsive is the noise. When the parameter α is close to 0 or 1, the density function may not be accurate because of numerical issues. On the other hand, the scale parameter γ behaves similarly to the variance of the Gaussian distribution. However, the α-stable distributions have unbounded variance. The only exception is for α=2 (the Gaussian case) when the α-stable distribution has a second-order moment.

The α-stable distributions have finite moments only for order lower than the parameter α. For instance, assuming a moment of order equal to *p*, the α-stable distribution has the following relation with α

(7) $$\begin{array}{ccc} \alpha <2, & \;E[X^{p}]\to \infty & \;\forall p\geq \alpha \\ \alpha <2, & \;E[X^{p}]<\infty & \;\forall 0\leq p<\alpha \\ \alpha =2, & \;E[X^{p}]<\infty & \;\forall p\geq 0 \end{array}$$

## 3. Experimental Setup

We use two sets of measurement equipment to collect audio data: (i) a low-cost setup with a ReSpeaker Core v1 (MT7688) board [26] using the Analog-to-Digital Converter (ADC) AC108 with four ADC delta-sigma, 48 kHz of sample rate, and 3.3 V voltage range, with four microphones connected to a Raspberry Pi 3 (model B) processor to collect and store the data; and (ii) a Data Acquisition (DAQ) NI-6361 from the National Instruments (National Instruments, Austin, TX, USA) as redundant equipment to validate the measures of low-cost setup with 16 bit resolution and 1 MS/s of sample rate. In this case, a Sony Vaio laptop (Core i3 processor, model PCG-61A11X, Vaio Corporation, Azumino, Japan) is used to receive the data from the DAQ.

The data is measured using the ReSpeaker at 48 kHz of sample rate, collecting 240,000 samples in 5 s. The microphone directional sensitivity is perpendicular from the source, and there is no obstruction or person between the source and the receivers. We acquired the audio signal for three situations:

1. Without audio source (only noise);
2. With a source emitting an audio tone of 1 kHz. This tone is produced by the Android App named Function Generator (keuwlsoft) [27], installed in a smartphone LG K10. The audio was reproduced by one of the channels of a JBL Flip 3 Portable Speaker (Harman International, Stamford, CT, USA). The microphones are set in a fixed position at 1.5 m from the audio source;
3. With a speech source. The speech source is from a person saying “this is just a test”. The microphones are set at the same position from the audio source.

**Figure 1:**
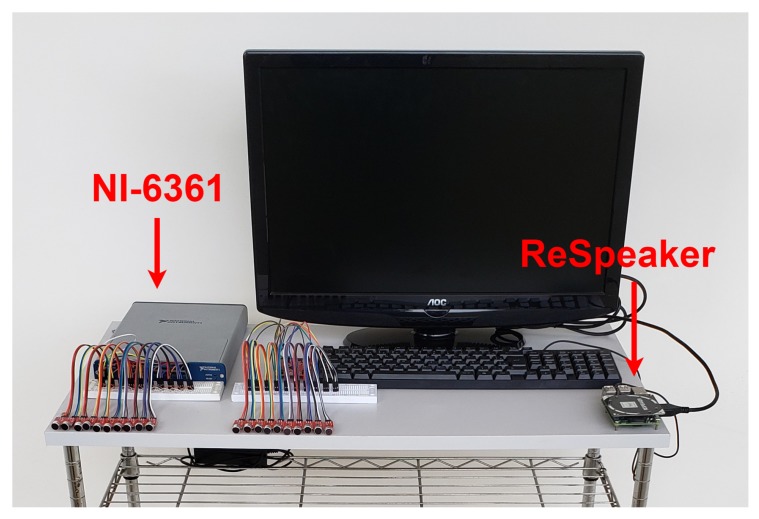
Experimental measurement setup.

The setup is mobile, allowing the instruments to move. The experimental setup is shown in Figure 1.

## 4. Experimental Results and Analysis

We acquired the data in three different scenarios:

1. **Indoor scenario:** A silent environment inside an empty auditorium;
2. **Hall scenario:** A mixed indoor/outdoor environment at the same auditorium hall;
3. **Outdoor scenario:** Outside the auditorium.

Investigations presented herein is organized as follows. Initially, we perform a time domain analysis of the signals to compare noise characteristics in the different scenarios. A power spectrum analysis is performed to verify the noise power level of each scenario. The impulsiveness is examined in the spectrum as well as its power level compared to signal power. Then, we show the Probability Density Function (PDF) fitting for Gaussian, GMM with two Gaussians and SαS models. We use the Root Mean Squared Error (RMSE) to measure the quality of the PDF fitting. After that, an investigation about the estimation window length is conducted to assess what is the influence of the number of samples and the windowing strategy to the distribution fitting. Finally, as the acoustic noise could exhibit a non-stationary behavior, we analyze the stationarity of the measured signal to ensure that the fitting is reliable.

### 4.1. Scenarios Analysis

**Figure 2:**
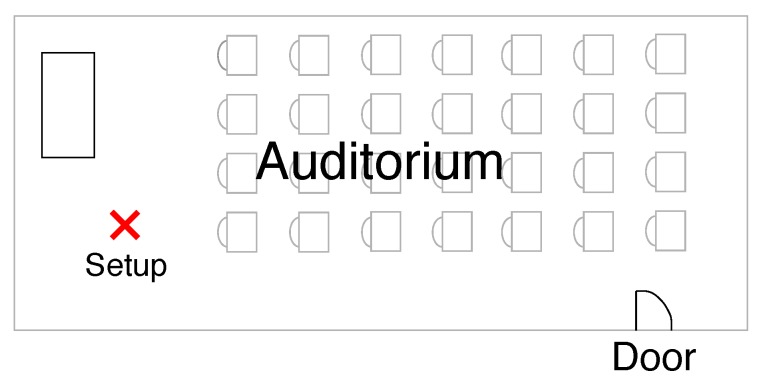
Indoor Scenario: Auditorium acoustically isolated without external audio noise.

Our first scenario is a silent auditorium illustrated in Figure 2. It is an environment with low-level noise, consequently presenting a high-quality audio signal.

**Figure 3:**
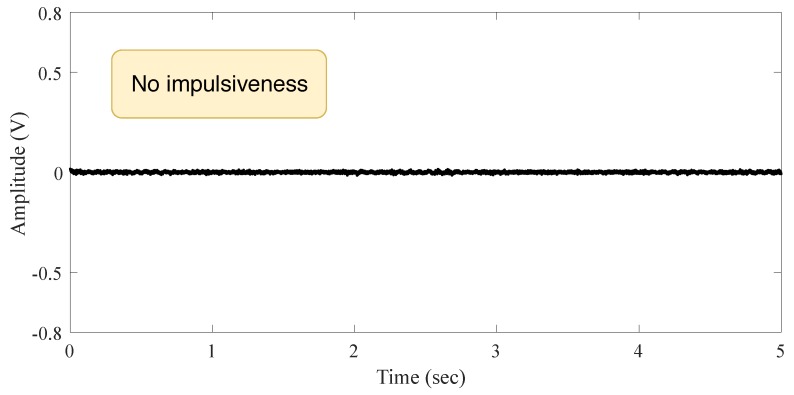
Signal behavior in the indoor scenario without audio source.

We labeled this environment as the **Indoor Scenario**. It is an auditorium acoustically isolated without external audio noise. This environment represents a place for conferences, presentations, with a low level of noise. The measured signal is shown in Figure 3 when there is no audio source. The signal does not present impulsiveness.

**Figure 4:**
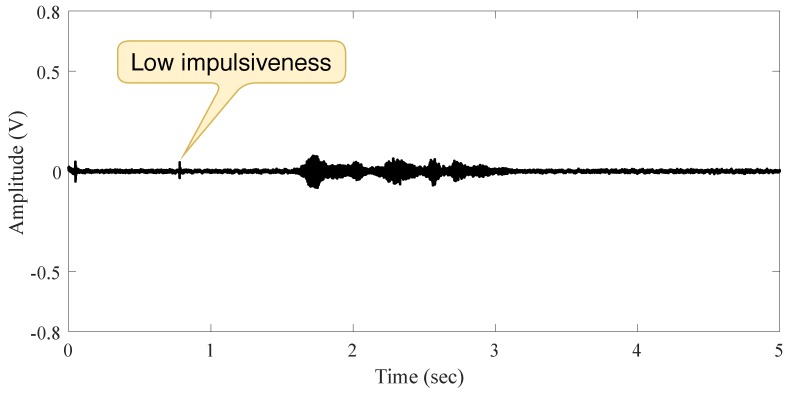
Signal behavior in the indoor scenario with a person moving and speaking.

The second measurement in indoor scenario is accomplished when a speech signal source is present, and the person intentionally made small moves before speaking. The measured signal exhibits a hardly ever impulsive noise (because of the person’s moving), as shown in Figure 4. This is evidence of the independence between noise and audio source as well as the low impulsiveness of noise in this scenario (low noise power compare to the signal power).

**Figure 5:**
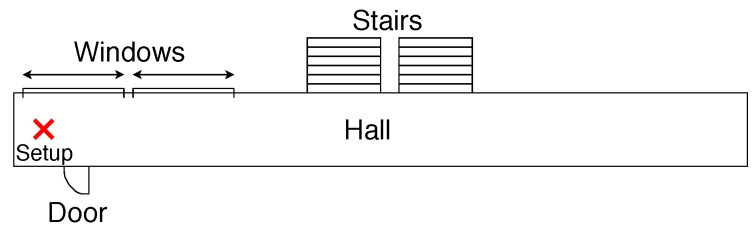
Hall Scenario: A mixed indoor/outdoor environment at auditorium hall.

We labeled the second environment as the **Hall Scenario**. It is a scenario noisier than the indoor, represented in Figure 5. Its mixed indoor/outdoor configuration is composed of two windows and two doors, allowing audio noise from wind. The measures were performed at night in the absence of noise from equipment as a Heating, Ventilation, and Air Conditioning.

**Figure 6:**
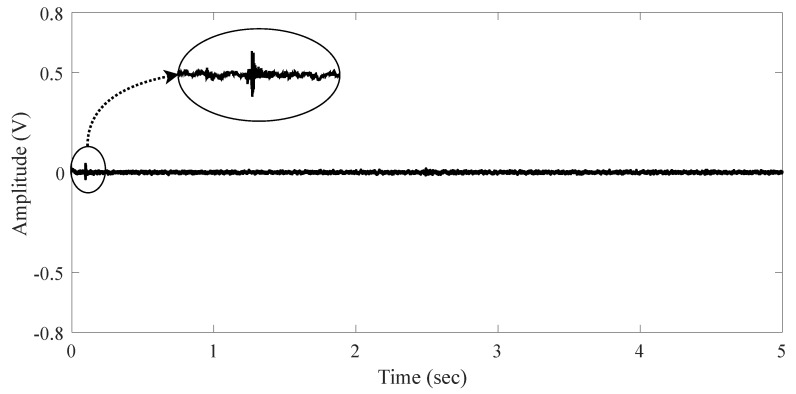
Signal behavior in the hall scenario without audio source.

Figure 6 presents the measured signal in the hall scenario, making evidence an infrequent impulsive noise, probably from some external source.

**Figure 7:**
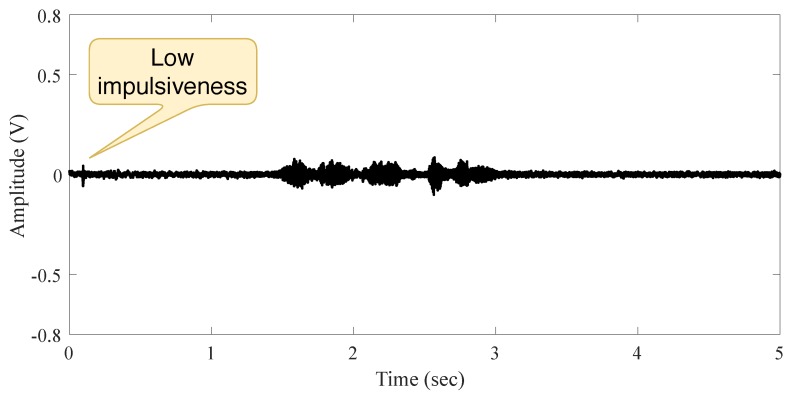
Signal behavior in the hall scenario with a person moving and speaking.

Figure 7 shows the signal when a person’s speech is present. As previously discussed in the indoor scenario, the independence between noise and audio source is observed, but now we see impulsiveness of noise due to some external source. However, this scenario has still low noise power compare to the signal power.

**Figure 8:**
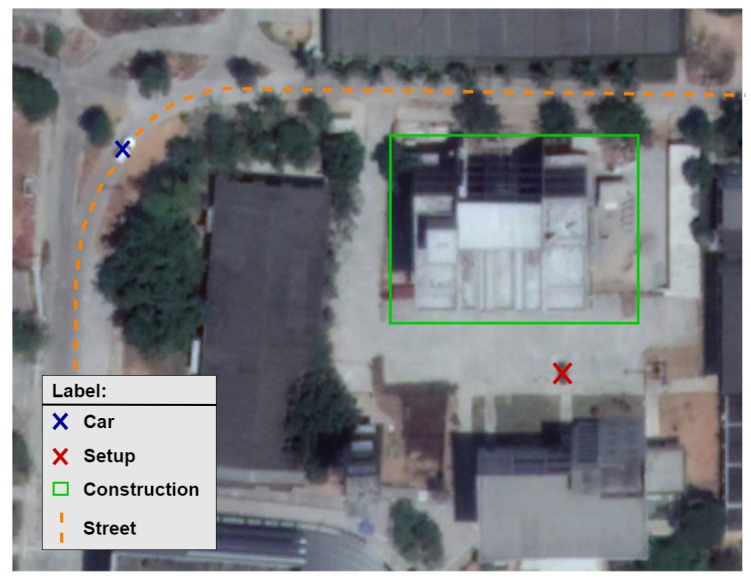
Outdoor scenario: Outside the auditorium with noise coming from the outside environment.

The third environment, named **Outdoor Scenario**, is illustrated in Figure 8. It is the noisiest one, with audio noise coming from the outside environment (building, traffic and crowd noise).

**Figure 9:**
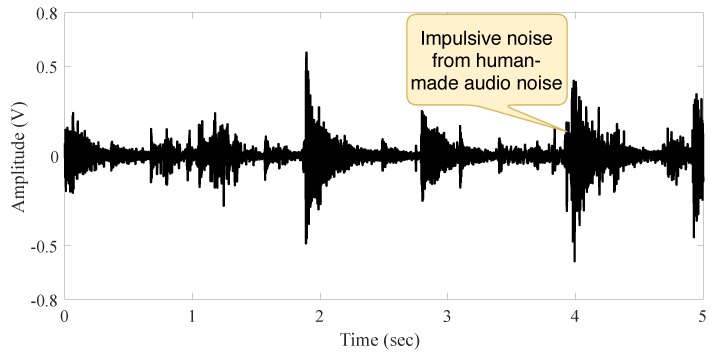
Signal behavior in the outdoor scenario without audio source.

**Figure 10:**
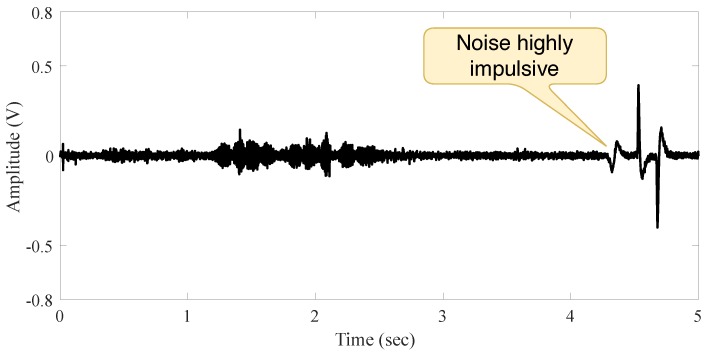
Signal behavior in the outdoor scenario with a person moving and speaking.

Figure 9 and Figure 10 present the signal in the outdoor environment without audio source and with a person’s speech, respectively. A careful observation of the signal behavior suggests that it does not have a constant second-order moment (its variance is time-dependent). This could happen as a consequence of different origins of noise, such as traffic, unknown speakers, crowds, wind, and human-made. We claim there is more impulsiveness if different noises sources are present.

### 4.2. Power Spectrum Density Analysis

**Figure 11:**
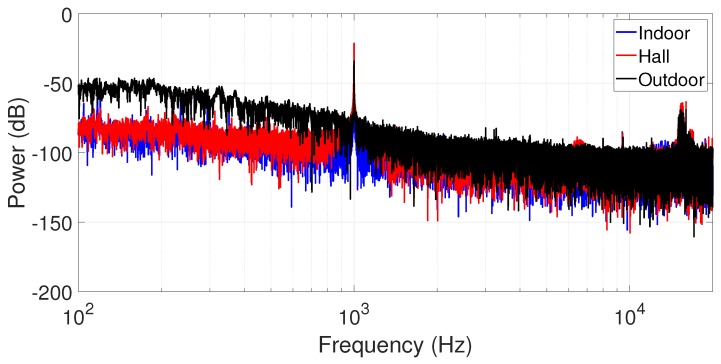
Power Spectrum Density of measured data in all scenarios (tone of 1 kHz of audio source).

We complete our analyses by characterizing the measured data in the frequency domain using the estimated Power Spectrum Density (PSD). Assuming the impossibility to measure the signal-to-noise ratio (SNR) of a signal subjected to a highly impulsive noise (because of its infinite variance), we estimate the PSD within a time window of 1500 samples for all scenarios. We highlighted a narrowband part of the spectrum from 100 Hz to 20 kHz and an audio source is the tone of 1 kHz at 1.5 m from the measurement point. Due to this proximity, the source is easily sensing, as shown in Figure 11.

### 4.3. PDF Fitting

Table 1 presents the estimation of the distribution’s parameters using 240,000 samples for each scenario without an audio source (only noise). From the estimation point of view, the mean of the Gaussian model is always zero, and the standard deviation is the parameter to be analyzed. Among the tested scenarios, the outdoor presents the highest Gaussian variance, indicating a lower SNR. As previously presented, the SαS model is characterized by α and γ parameters. The lowest values of such parameters are estimated for the outdoor scenario, evidencing a high impulsiveness level and a lower dispersion, respectively. The α value of about 1.27 of outdoor scenario indicates a non-Gaussian noise with frequent impulsiveness characteristics. We assume two Gaussian distributions for GMM model. In the indoor scenario, the GMM does not have zero mean. In this case, the two Gaussians are not enough to fit the data. In the outdoor scenario, variances of two Gaussians are very different, capturing the impulsiveness characteristic of the noise.

**Figure 12:**
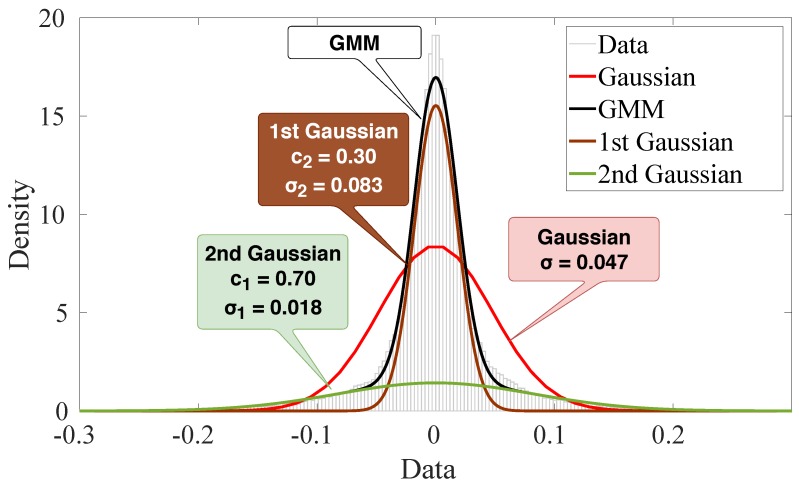
Illustration of Gaussian mixture model fitting with two Gaussians (data from the outdoor scenario in a time window with severe impulsiveness).

**Figure 13:**
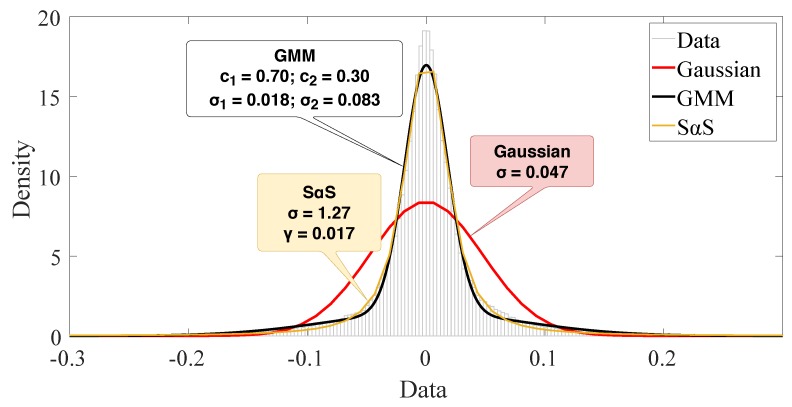
Illustration of PDF fitting for all models (data from the outdoor scenario in a time window with severe impulsiveness).

For illustrative purposes, we build the Figure 12 and Figure 13 for 1500 samples of outdoor scenario in a time window with severe impulsiveness (a lower SαS’s α value). Figure 12 presents a comparison of the Gaussian and GMM PDFs with the estimated parameters as well as the two Gaussians’ PDF of GMM. As the GMM is the linear combination of two Gaussians weighted by $c_{1}$ and $c_{2}$, each Gaussian has its different variances $\sigma_{1}$ and $\sigma_{2}$ that can be associated to different sources of noise. Although the individual Gaussians of GMM has a poor fit due to heavy tails of the data, the GMM may reach a better fit using its two Gaussian components.

Figure 13 shows a similar plot including the SαS fitting. The PDF is computed using the direct integration method, as described in [25]. We also visualize the significant error from the Gaussian model fitting. However, it is very hard to precise if GMM is better than the SαS model.

**Figure 14:**
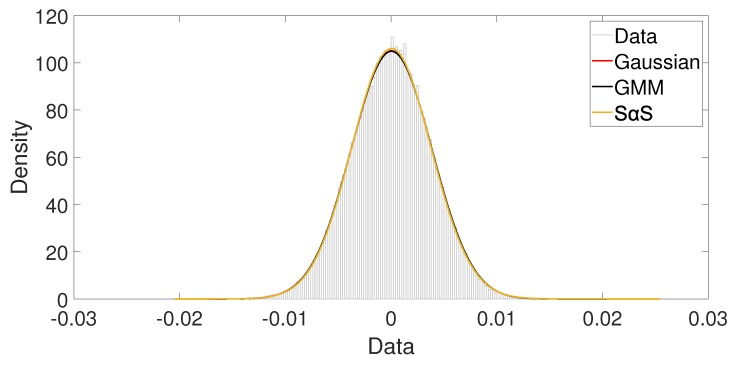
Indoor scenario: Visual comparison among Gaussian, GMM and SαS PDF fitting.

**Figure 15:**
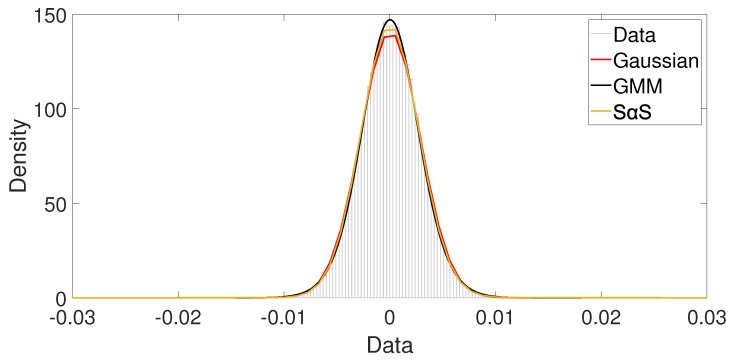
Hall scenario: Visual comparison among Gaussian, GMM and SαS PDF fitting.

This behavior is also observed when we draw similar plots for indoor and hall scenarios, Figure 14 and Figure 15, respectively. In the indoor scenario, no difference is noticed among the models. The low impulsiveness causes a better fit from the Gaussian model as shown in Figure 14. The two components from the GMM model have similar behaviors; probably they are modeling the same class of noise. The α from SαS model has the value close to 2, indicating that the model is similar to a Gaussian model. In the hall scenario, all models also have similar performance, as shown in Figure 15. However, compared to the indoor case, the α can reach slightly lower values, indicating a higher impulsiveness condition. Therefore, as mentioned before, we can not determine the best model by only a visual inspection.

Finally, we evaluate numerically the quality of fit by the Root Mean Square Error (RMSE). Results showed in Table 2 indicates the SαS model has the best fit for all scenarios. The GMM reaches a better fit than the Gaussian model in the hall and outdoor scenarios, where the impulsiveness exists. In the indoor scenario, due to the absence of impulsiveness, the Gaussian model has no difficulty to fit data, although the SαS has slightly better RMSE.

**Figure 16:**
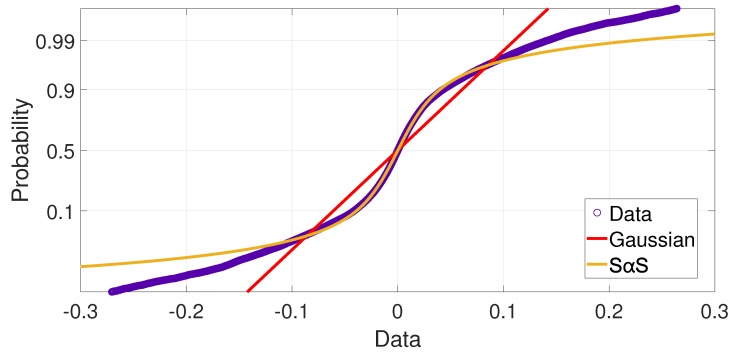
Outdoor scenario: Comparison between the data distribution and the estimated Gaussian and SαS models.

As expected, the simplest Gaussian model has not succeeded to fit well the data with impulsiveness due to its unbounded variance. We conclude that Gaussian is not a suitable model for this data, whereas the GMM has better performance. Finally, the SαS model has the best fitting performance, as a result of accurate modeling of the heavy tails. Figure 16 confirms this thought by showing the comparison of the cumulative distribution of data and the Cumulative Distribution Function (CDF) of Gaussian and SαS models (with the estimated parameters of the Table 1). The plots are for the outdoor scenario where the low value of α suggests high impulsiveness and a non-Gaussian behavior [8].

### 4.4. Sample Number Analysis

A crucial issue of signal estimation is the stationarity assumption [28]. If data is non-stationary, we can not affirm that the estimated parameters (e.g., mean, variance and autocorrelation) do not change over time. However, several researchers persist with stationarity assumption because the problems are mathematically easier to model. Initially, we present an investigation about the number of samples and the windowing strategy. In this section, we start the discussions about stationarity, and in the Section 4.5, we provide a more statistically rigorous hypothesis test to legitimate the estimated model for each scenario.

**Figure 17:**
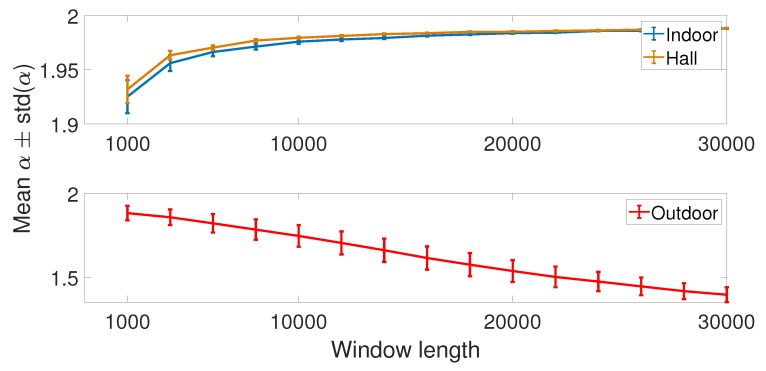
Variance of the estimated α parameter versus the window length.

First, we show in Figure 17 the variance of the α values (from SαS model) estimated for the whole set of 240,000 samples. We split samples in time windows of fixed size and evaluate the variances of the estimated α inside window sizes from 1000 to 30,000. Looking at the results for indoor and hall scenarios, we see that longer the window size is, the more stable is the estimation (the lower the variance). However, for the outdoor scenario, this behavior is only observed for more extended window sizes (longer than shown in the Figure 17, suppressed for better visualization of curves). Comparing the results for the three scenarios, we can choose a window length of 1500 samples for the parameter analysis. This window provides a trade-off of low variance (estimation stability) and the window size. From a system point of view, a long window size could reflect higher signaling cost or latency.

**Figure 18:**
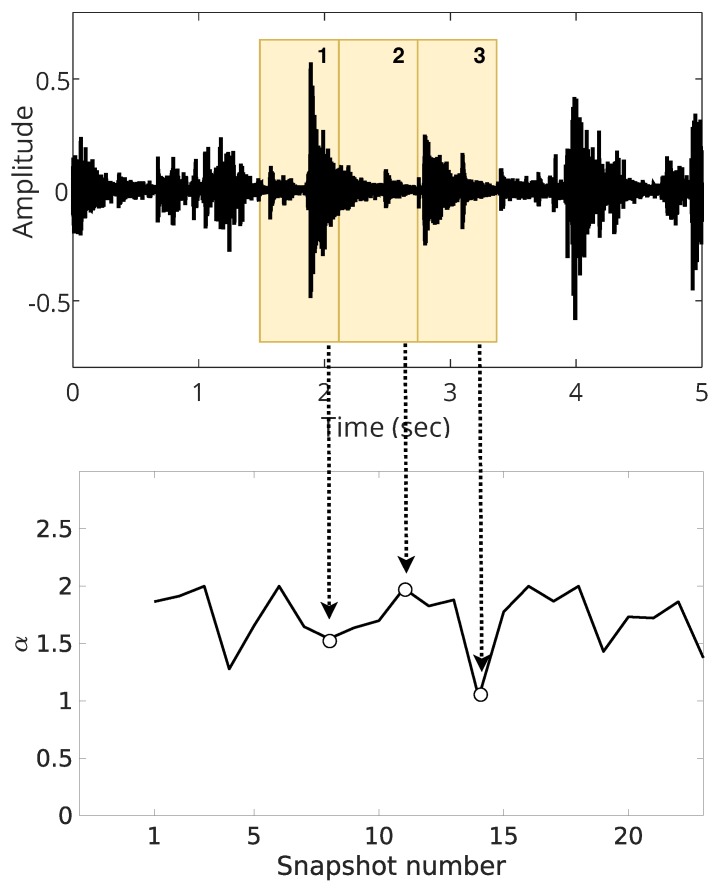
Illustration of non-overlapped estimation.

**Figure 19:**
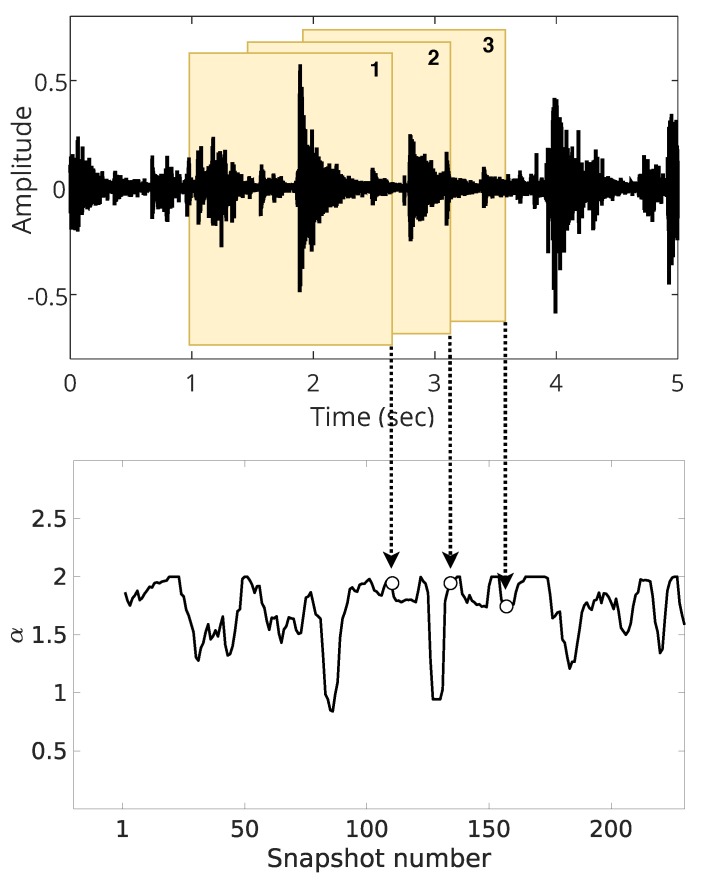
Illustration of overlapped estimation.

Secondly, only with illustrative purposes, we present the difference between overlapped and non-overlapped estimation in Figure 18 and Figure 19. When we use a non-overlapped estimation, shown in Figure 18, the estimated parameters are much smoother than overlapped estimation, illustrated in Figure 19.

In both cases, the window size is 10,000 samples, but we overlapped 1000 samples (10% of the window) for the estimation in Figure 19. The objective is to show that, due to the different number of snapshots (24 for the non-overlapped and 240 for the overlapped case), the overlapped estimation better captures the variations of the estimated parameters. Thus, we use the overlapped window technique to get better analysis, exploring the trade-off between low window length and high resolution estimation.

**Figure 20:**
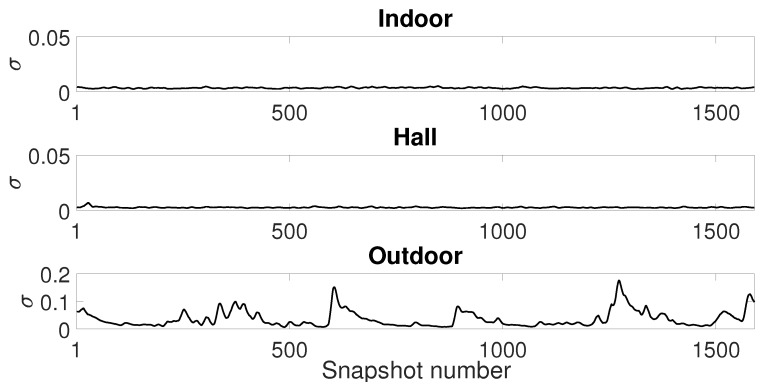
Gaussian model estimation: Sample window of 1500 with 10% of windows overlapping.

**Figure 21:**
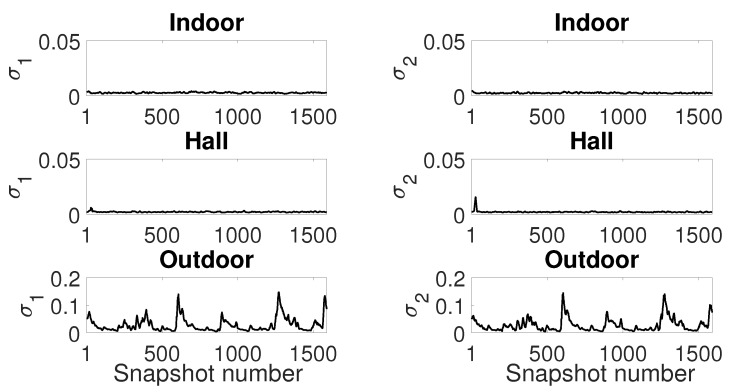
GMM model estimation: Sample window of 1500 with 10% of windows overlapping.

**Figure 22:**
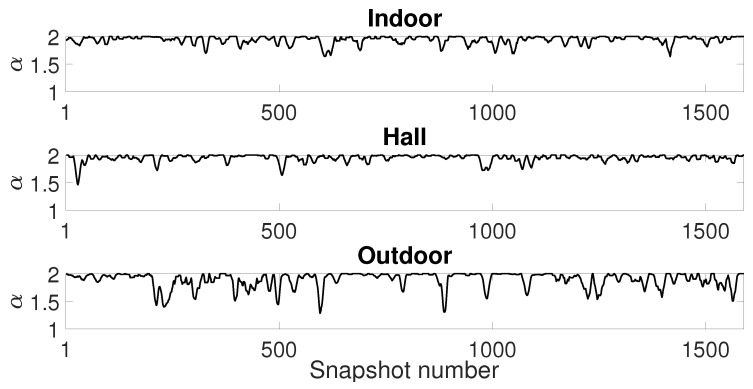
SαS model estimation: Sample window of 1500 with 10% of windows overlapping.

Now, we analyze the estimation stability for the Gaussian (Figure 20), GMM (Figure 21) and SαS (Figure 22) models for all scenarios. We plot the estimated parameters of 1590 snapshots for a window size of 1500 with 10% of window overlapping (this is the total number of snapshots with 240,000 samples, window size of 1500 and 150 overlapped samples, calculated as (240,000−1500)/150=1590 snapshots). We show the parameter estimation for the two Gaussians of GMM model (Figure 21).

Analyzing all figures, while the indoor and hall scenarios present reasonable stability on parameter estimation, especially considering Gaussian and GMM models, the outdoor scenario presents an estimation with significant variance.

Finally, looking at the parameters for α-stable model (Figure 22), one can confirm that the outdoor scenario has a highly impulsive noise due to the α values near to 1. We may conclude that α values far from 2 indicate a poor fitting of Gaussian model and noise data. It suggests that the variance from Figure 20 is not enough to describe the noise data, especially from the outdoor scenario.

As discussed previously discussed, from a system perspective, short window size produces less signaling cost and latency. Therefore, using a large window, the estimated parameters converge to a specific value, and in a short window, the parameters vary with the time because the estimation reflects the inclusion or not of impulsiveness events in the measured signal.

### 4.5. Stationarity Test

There are other ways to verify the stationarity of a time series. The autocovariance may quantify the degree of association between two points separated by a lag. Low values of autocovariance indicate no similarity with a delayed version of itself [28].

**Figure 23:**
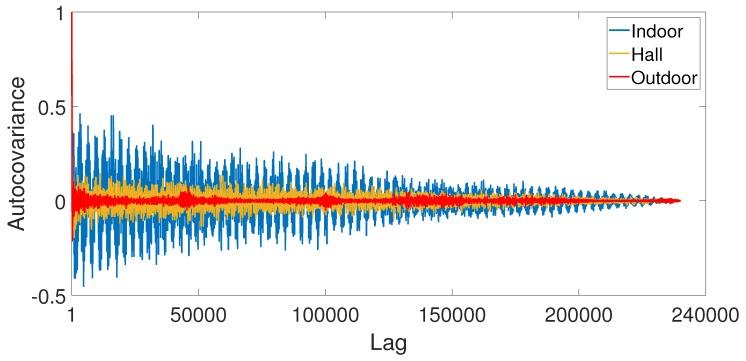
Autocovariance of the measured data for all scenarios (with no audio source).

Figure 23 presents the autocovariance of the measured signal for all scenarios (without audio source). Thus, it seems reasonable to consider that the outdoor scenario represents non-stationary behavior due to the uncorrelated signal with itself. On the other hand, the indoor scenario has high values of autocovariance, representing a stationary behavior.

Now, we use a stationarity test, named Kwiatkowski, Phillips, Schmidt, and Shin (KPSS) test for a unit root in the univariate time series [29]. In this statistic test, $H_{1}$ indicates rejection of the stationary null in favor of the unit root alternative, and $H_{0}$ indicates a failure to reject the stationary null. We apply the test for all scenarios and evaluated if the signal is a unit root process, i.e., stationary, against the alternative that there is no unit root.

Table 3 shows the KPSS for our measured data. The indoor and hall environments reject the stationary null in favor of the unit root alternative with low standard error. However, the outdoor environment test fails to reject the null hypothesis that the signal is stationary, as expected. The test statistic *p*-value reaches the maximum value of 0.10, and the test statistics, computed by ordinary least squares regression, is lower than the other scenarios.

## 5. Conclusions

We present a complementary study about the characterization of non-Gaussian impulsive noise. Investigations are based on collected data in three scenarios, representing real-life places for conferences and presentations. The acoustic signal is analyzed from undesirable interference caused by noise from vehicles, crowds, wind, machines and human-made audio noise. We also evaluate the situations without audio source (only noise), with a source emitting an audio tone of 1 kHz, and finally with a speech source.

We evaluate the density model fitting, the number of samples and two windowing strategies at the light of model complexity and accuracy. Stationarity is evaluated by more than one methodology, helping the discussion of our fitting reliability.

For scenarios with critical impulsiveness, non-Gaussian models have better goodness of fit. The SαS model is the best model, but unnecessary when the signal has low-level of impulsiveness. The GMM is an alternative to SαS model due to its capacity to model noises from different sources. Therefore, the models may be chosen based on the degree of impulsiveness present in the acoustic channel, i.e., in the target scenario.

## Figures and Tables

**Table 1:** Parameters estimated for Gaussian, Gaussian mixture model, and α-stable models.

	Gaussian	SαS		GMM					
Scenario	σ	α	γ	$c_{1}$	$\mu_{1}$	$\sigma_{1}$	$c_{2}$	$\mu_{2}$	$\sigma_{2}$
**Indoor**	0.003793	1.9817	0.3390	0.5934	−0.0009358	0.003618	0.4066	0.001466	0.003584
**Hall**	0.002837	1.9764	0.3513	0.6165	0	0.002402	0.3835	0	0.003422
**Outdoor**	0.04743	1.2669	0.005058	0.7043	0	0.01809	0.2957	0	0.08264

**Table 2:** Performance of data probability density function fitting by the root mean square error.

	Gaussian	GMM	Sα-S
**Indoor**	1.1936	1.1988	1.1925
**Hall**	1.0221	0.6757	0.5817
**Outdoor**	1.4969	0.2820	0.2072

**Table 3:** Stationarity Kwiatkowski, Phillips, Schmidt, and Shin (KPSS) test for all scenarios (with no audio source).

	Indoor	Hall	Outdoor
**Hypothesis test**	$H_{1}$	$H_{1}$	$H_{0}$
***p*-Value**	0.01	0.01	0.10
**Test statistics**	3.6629	7.5457	0.0443
**Standard error**	0.0038	0.0028	0.0474
